# Compressive Sensing Hyperspectral Imaging by Spectral Multiplexing with Liquid Crystal

**DOI:** 10.3390/jimaging5010003

**Published:** 2018-12-22

**Authors:** Yaniv Oiknine, Isaac August, Vladimir Farber, Daniel Gedalin, Adrian Stern

**Affiliations:** Department of Electro-Optical Engineering, Ben-Gurion University of the Negev, Beer-Sheva 84105, Israel

**Keywords:** compressive sensing, hyperspectral imaging, multiplexing system, liquid crystal, three-dimensional imaging, integral imaging, remote sensing, point target detection, CS-MUSI

## Abstract

Hyperspectral (HS) imaging involves the sensing of a scene’s spectral properties, which are often redundant in nature. The redundancy of the information motivates our quest to implement Compressive Sensing (CS) theory for HS imaging. This article provides a review of the Compressive Sensing Miniature Ultra-Spectral Imaging (CS-MUSI) camera, its evolution, and its different applications. The CS-MUSI camera was designed within the CS framework and uses a liquid crystal (LC) phase retarder in order to modulate the spectral domain. The outstanding advantage of the CS-MUSI camera is that the entire HS image is captured from an order of magnitude fewer measurements of the sensor array, compared to conventional HS imaging methods.

## 1. Introduction

Hyperspectral (HS) imaging has gained increasing interest in many fields and applications. These techniques can be found in airborne and remote sensing applications [1,2,3,4], biomedical and medical studies [5,6,7], food and agricultural monitoring [8,9,10], forensic applications [11,12], and many more. The HS images captured for these applications are usually arranged in three-dimensional (3D) datacubes, which include two dimensions (2D) for the spatial information and one additional dimension (1D) for the spectral information. With a 2D spatial domain of megapixel size and with the third (spectral) dimension typically containing hundreds of spectral bands, the HS data is usually huge. Consequently, its scanning, storage and digital processing is challenging.

**Figure 1:**
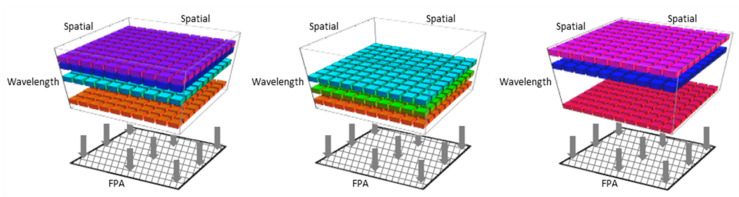
Spectral multiplexing. The figure represents three different examples of spectral multiplexing. Each sub-figure illustrates multiplexing of a few spectral bands onto a FPA.

Studies have shown that the huge HS datacubes are often highly redundant [13,14,15,16,17] and, therefore, very compressible or sparse. This gives the incentive to implement Compressive Sensing (CS) theory in HS systems. CS is a sampling framework that facilitates efficient acquisition of sparse signals. Numerous techniques that employ the CS framework for spectral imaging [18,19,20,21,22,23,24,25,26] have been proposed in order to reduce the scanning efforts. Most of these techniques involve spatial–spectral multiplexing, which is suitable for CS; however, this multiplexing inevitably impairs both the spatial and spectral domains. In References [27,28], we introduced a novel CS HS camera dubbed the Compressive Sensing Miniature Ultra-Spectral imaging (CS-MUSI) camera. The CS-MUSI camera overcomes the impairment in the spatial domain by performing only spectral multiplexing without any spatial multiplexing. Figure 1 provides a schematic description of HS datacubes that undergo only spectral multiplexing. The figure presents three examples of a HS datacube modulated at three different exposures. Different spectral multiplexing is obtained by applying different conditions on the modulator. The spectrally multiplexed data is ultimately integrated by a focal plane array (FPA).

Within the framework of CS, the CS-MUSI camera can reconstruct HS images with hundreds of spectral bands from only spectrally multiplexed shots, numbering an order of magnitude less than would be required using conventional systems. Furthermore, the CS-MUSI camera benefits from high optical throughput, small volume, light weight, and reduced acquisition time. In this paper, we review the evolution of the CS-MUSI camera and its different applications.

In this article, we first review the innovative concept behind our CS-MUSI camera. We describe spectral multiplexing within the CS framework using a liquid crystal (LC) phase retarder, and expand on the sensing and reconstruction processes. In the following, we outline the optical setup of the CS-MUSI camera and its realization. Lastly, we describe different possible applications for the CS-MUSI camera, including HS staring imaging, HS scanning imaging, 4D imaging, and target detection tasks.

## 2. Spectral Modulation for CS with LC

**Figure 2:**
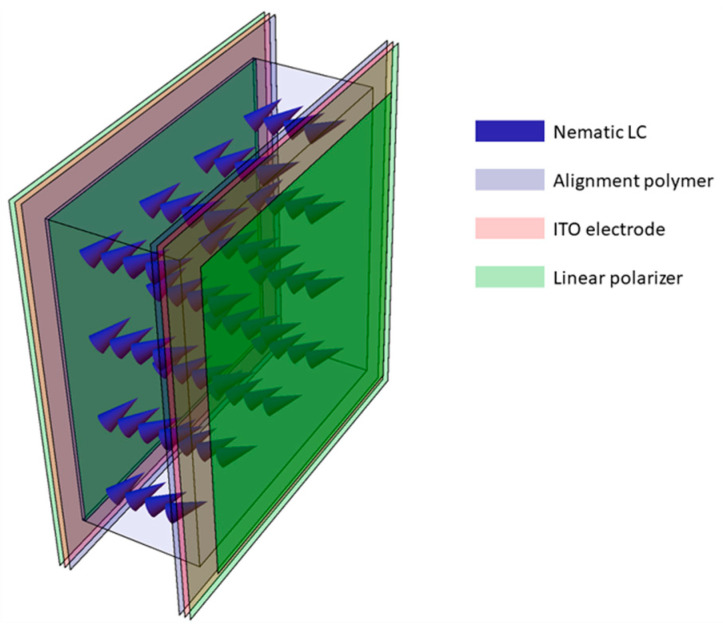
LC cell phase retarder. The LC phase retarder is made of a Nematic LC layer (blue arrow) sandwiched between two glass plates and two linear polarizers (green layers). The glass plates are coated with Indium Tin Oxide (ITO, pink layers) and a polymer alignment layer (purple layers).

The core of the CS-MUSI camera is a single LC phase retarder (Figure 2), which we designed to work as a spectral modulator that is compliant with the CS framework [27]. By using the LC phase retarder, the signal multiplexing is accomplished entirely in the spectral domain, and no spectral-to-spatial transformations are required. The LC phase retarder is built by placing a LC cell between two polarizers. The spectral transmission is controlled by the voltage applied on the LC cell, causing variations in the cell’s birefringence, which, in turn, cause refractive index changes. For the case where the optical axis of the LC cell is at 45° to two perpendicular polarizers, the spectral transmission response of the LC phase retarder can be described by [29]:

(1) $$\phi_{\mathrm{LC}}\left(\lambda ,V_{i}\right)=\frac{1}{2}-\frac{1}{2}\cos\left(\frac{2\pi ∆n(V_{i})d}{\lambda }\right),$$

where $∆n(V_{i})$ is the birefringence produced by voltage $V_{i}$, *d* is the cell thickness and λ is the wavelength. The voltage applied to the LC cell is an AC voltage, usually in the form of sine or square wave and with a typical frequency in the order of kHz.

**Figure 3:**
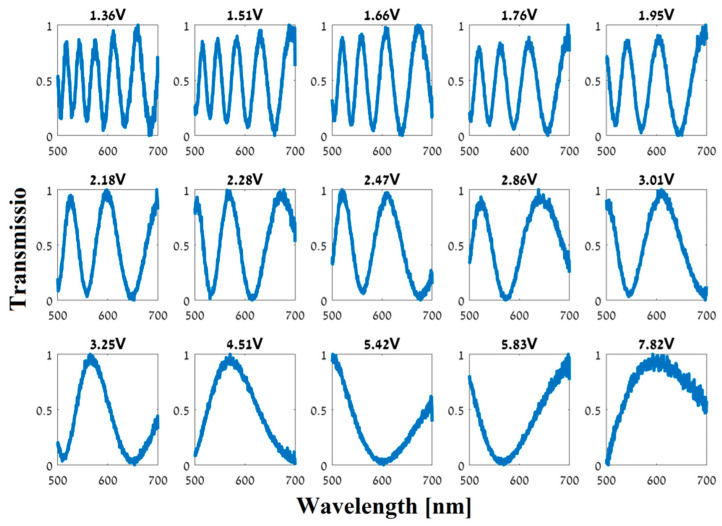
Measured spectral responses (intensity transmission vs. wavelength in nm) of the fabricated LC phase retarder. Each graph represents the spectral modulation with a different voltage applied on the LC cell (15 different voltages).

We designed the LC cell to have a relatively thick cavity (tens of microns) to facilitate modulation over a broad spectrum with oscillatory behavior, as can been seen in the different spectral responses presented in Figure 3. The figure presents 15 plots of 15 different measured spectral responses for different AC voltages (2 kHz square wave) applied on the LC cell. It can be noticed that as the voltage decreases [higher birefringence, $∆n(V_{i})$], the number of peaks in the spectral transmission graphs rises. Theoretically, these spectral responses should follow expression (1), spanning the entire range from 0 to 1 for all the wavelength range. However, in practice, we can see that the modulation depths in Figure 3 are not equal for all the peaks. This is due to the quality of the polarizers.

**Figure 4:**
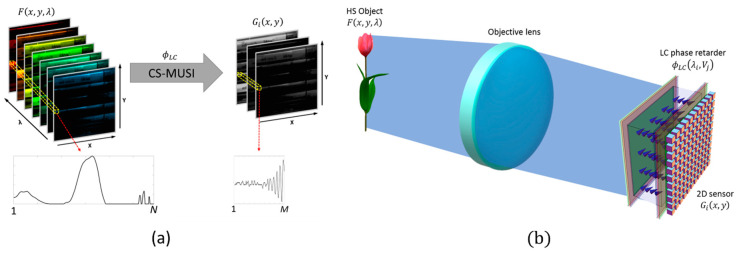
(**a**) CS-MUSI acquisition process. (**b**) CS-MUSI optical scheme diagram. The HS object F(x,y,λ) is modulated according to $\phi_{\mathrm{LC}}\left(\lambda ,V_{i}\right)$, yielding the multiplexed measurement $G_{i}(x,y)$.

The acquisition process and the optical scheme of the CS-MUSI camera are shown in Figure 4. The spatial–spectral power distribution of the HS object, F(x,y,λ), is modulated by the LC phase retarder transmittance function, $\phi_{\mathrm{LC}}\left(\lambda ,V_{i}\right)$ (the graph from Figure 3). The *i*’th modulated spectral signal is spectrally multiplexed and integrated at each pixel in the 2D sensor array, which gives the encoded measurements:

(2) $$G_{i}(x,y)=\int \phi_{\mathrm{LC}}\left(\lambda ,V_{i}\right)F(x,y,\lambda )d\lambda .$$

As the sensor samples discrete values, and for CS analysis and reconstruction purposes, it is more convenient to present the sensing process from Equation (2) in a matrix-vector format. Let us denote the spectral signal with *N* spectral bands by $f\in {ℜ}^{N\times 1}$ and the multiplexed measured spectral signal with *M* entries by $g\in {ℜ}^{M\times 1}$. By using these vectors, the measurement process can be described by:

(3) g=Φf,

where $\Phi \in {ℜ}^{M\times N}$ represents the CS sensing matrix. Compressive sensing [22,30,31,32] provides a framework to capture and to recover signals from fewer measurements than required by the Shannon-Nyquist sampling theorem (i.e., *M* < *N*). The CS framework relies on three main ingredients. First, the sparsity of the signal, namely, the spectral signal with *N* spectral bands can be expressed by f=Ψα, where α is a *K*-sparse vector (containing *K* << *N* non-zero elements) and Ψ is the sparsifying operator. In accordance with the CS framework, Equation (3) can be written as:

(4) g=ΦΨα=Ωα,

where Ω=ΦΨ. Second, the CS systems needs an appropriate sensing design, which is represented by the system sensing matrix, Φ. Third, the CS framework relies on the existence of an appropriate reconstruction algorithm, upon which we will expand in the next section.

## 3. Reconstruction Process

The CS-MUSI camera was designed in accordance with the CS framework. Consequently, the captured data is a compressed version of the scene’s HS datacube. Therefore, a reconstruction process that solves Equation (4) should be performed. Over the years, several CS algorithms have been developed [32,33,34,35,36,37] in order to recover the original signal. A common class of these reconstruction algorithms solve the $\ell_{2}-\ell_{1}$ minimization problem:

(5) $$\tilde{\alpha }=\underset{\alpha }{\arg\min}\left\{\frac{1}{2}{\left\|g-\Phi \Psi \alpha \right\|}_{2}^{2}+\tau {\left\|\alpha \right\|}_{1}\right\}.$$

where $\tilde{\alpha }\in {ℜ}^{N\times 1}$ is the estimated *K*-sparse signal, *τ* is a regularization parameter and ${\left\|\cdot \right\|}_{p}$ is the $l_{p}$ norm. The algorithms recover the original signal by using the known system sensing matrix, Φ, and the signal sparsifying operator, Ψ [22,37,38,39]. The sparsifying operator can be a mathematical transform (DCT, Wavelet, Curvelets etc.) or a learned dictionary; the latter has shown promising results [40] and will be described in the next subsection. Common algorithms developed to solve Equation (5) are TwIST [33], GPSR [34], SpaRSA [35], TVAL3 [36], etc.

### Dictionary for Sparse Representation

The first ingredient that the CS framework relies on is the sparsity of the signal. The sparser the representation of the signal is, the better the CS reconstruction algorithms perform. We found [40] that using a learned dictionary [38] as the sparsifying operator can significantly improve the reconstruction accuracy in comparison to using a mathematical basis. In addition, using a dictionary can reduce both the time and the number of measurements required in order to reconstruct the original signal. A dictionary is a learned sparsifier from exemplars. In order to be able to use a dictionary in CS algorithms, a preprocessing stage has to be performed, and it is done only once.

First, a large database of spectral signals, $S\in {ℜ}^{N\times N_{S}}$, is collected. This database contains $N_{S}$ spectra with *N* spectral bands:

(6) $$S=\left[s_{1},s_{2},\ldots ,s_{N_{S}}\right],$$

where $s_{i}$ is the *i*’th spectrum in the database. Then, an over-complete spectral dictionary with $N_{d}$ atoms to train, $\Psi_{d}\in {ℜ}^{N\times N_{d}},$ is created by applying a dictionary-learning algorithm, such as the K-SVD algorithm [38], to the spectral database **S**:

(7) $$\Psi_{d}=\text{K-SVD}\left\{S\right\}.$$

$\Psi_{d}$ is a dictionary that relates the spectral data **f** to its sparse representation, $f=\Psi_{d}\alpha$. Each column of $\Psi_{d}$ is referred to as an atom of the dictionary. Therefore, the spectrum **f** can be viewed as a linear combination of atoms in $\Psi_{d}$ according to weights in α. Based on Equation (7), a corresponding system dictionary $\Omega_{d}\in {ℜ}^{M\times N_{d}}$ is created by the inner products of the spectral dictionary with the CS-MUSI sensing matrix, Φ:

(8) $$\Omega_{d}=\Phi \Psi_{d}.$$

After the dictionary has been prepared, it can be used as the sparsifying operator to reconstruct the original spectral signal by finding the estimated atom weights vector, ${\tilde{\alpha }}_{d}$. These weights could be found, for example, by the $l_{2}-l_{1}$ minimization problem from Equation (5) that becomes:

(9) $${\tilde{\alpha }}_{d}=\underset{\alpha }{\arg\min}\left\{\frac{1}{2}{\left\|g-\Omega_{d}\alpha \right\|}_{2}^{2}+\tau {\left\|\alpha \right\|}_{1}\right\}.$$

Once the atom weights are found the original spectral signal is estimated by applying the atom weights on the spectral dictionary we created, $\Psi_{d}$ (Equation (7)):

(10) $$\tilde{f}=\Psi_{d}{\tilde{\alpha }}_{d}.$$

For more details on the utilization of dictionaries for CS-MUSI data reconstruction and its advantages over other sparsifiers, the reader is referred to [40].

## 4. Compressive Hyperspectral and Ultra-Spectral Imaging

**Figure 5:**
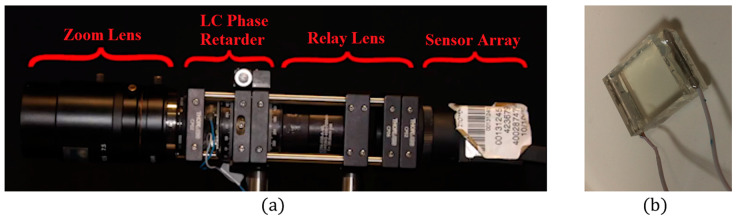
(**a**) Realization of the CS-MUSI camera. (**b**) In-house manufactured LC cell.

The CS-MUSI camera we built is shown in Figure 5a. It is slightly different from the optical setup that was designed and presented in Figure 4, but is optically equivalent. It has a 1:1 optical relay that projects the LC plane onto a 2D sensor array, thus avoiding the need to attach the LC cell to the 2D sensor array. A LC cell is placed in the image plane of a zoom lens. The light transmitted through the LC cell is conjugated to a sensor array using a 1:1 relay lens. The optical sensor of the camera is a uEye CMOS UI-3240CP-C-HQ with 1280 × 1024 pixels, with a pixel size of 5.3 μm × 5.3 μm and 8-bit grayscale level radiometric sampling. The camera LC cell from Figure 5b was manufactured in-house and has a cell gap of approximately 50 μm and a clear aperture of about 8 mm × 8 mm. The LC cell was fabricated from two flat glass plates coated with Indium Tin Oxide (ITO) and a polymer alignment layer. The cavity is filled with LC material E44 (Merck, Darmstadt, Germany). Together with two linear polarizers on both sides of the LC cell, the LC phase retarder is created (Figure 2).

### 4.1. Camera Calibration

**Figure 6:**
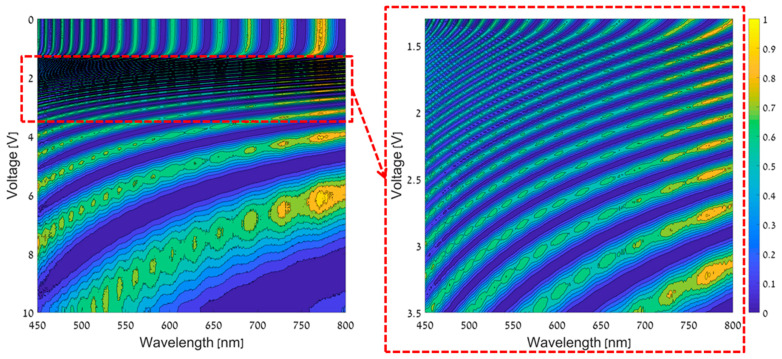
CS-MUSI spectral response map for voltages from 0 V to 10 V (**left map**) and a zoom in on the area where the voltages are from 1.3 V to 3.5 V (**right map**).

The theoretical expression in Equation (1) cannot be used directly in order to obtain the system-sensing matrix, because the dependence of the birefringence on the voltage and the material dispersion are unknown. Consequently, a calibration process in which the spectral responses of the camera were precisely measured, was performed once. Using a point light source as the object and by replacing the sensor of the camera with a commercial high-precision grating spectrometer, the spectral responses of the camera were measured. The calibration process was performed by using a halogen light source with a pre-measured spectrum and by applying voltages from 0 V to 10 V on the LC cell with steps of 2 mV. Figure 6, presents the CS-MUSI system’s spectral response map that was measured in the calibration process.

The system sensing matrix, Φ, is obtained by selecting *M* rows from the CS-MUSI system’s spectral response map (Figure 6, left). This is done by using a sequential forward floating selection method [28,41] that aims to achieve a highly incoherent set of measurements.

### 4.2. Staring Mode

**Figure 7:**
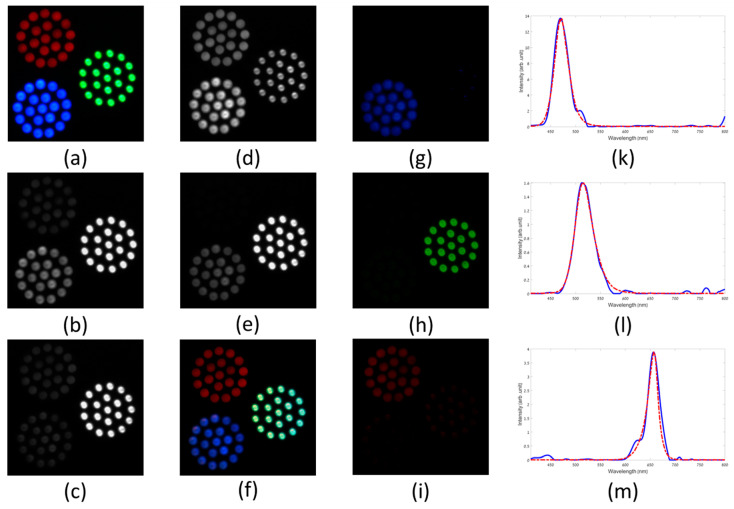
Staring mode reconstruction result of three LED arrays. (**a**) RGB color image of three LED arrays that were used as objects to be imaged with CS-MUSI. (**b**–**e**) Representative single exposure images for LC cell voltage of 0 V, 5.8373 V, 7.6301 V and 8.6552 V, respectively. (**f**) RGB representation of the reconstructed HS image (700 × 700 pixels× 391 bands). (**g**–**i**) Reconstructed images at 460 nm, 520 nm and 650 nm, respectively. (**k**–**m**) Spectrum reconstruction for three points in the HS datacube and comparison to the measured spectra of the three respective LEDs with a commercial grating-based spectrometer.

**Figure 8:**
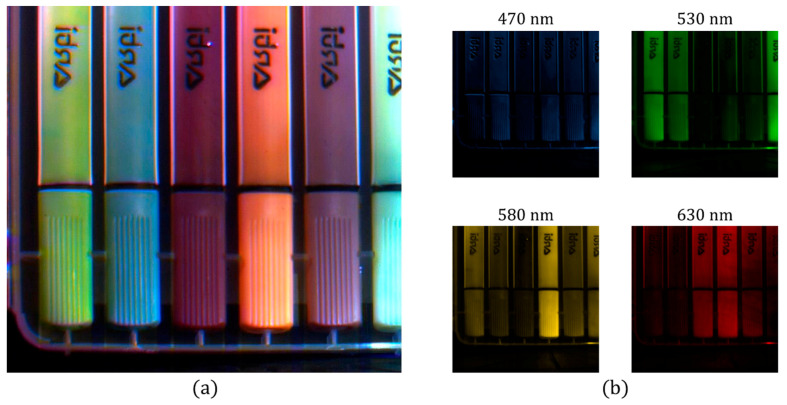
Staring mode reconstruction result of six different markers. (**a**) RGB representation of the reconstructed HS image (800 × 900 pixels × 1171 bands). (**b**) Four reconstructed images at four different wavelengths (470 nm, 530 nm, 580 nm, and 630 nm).

**Figure 9:**
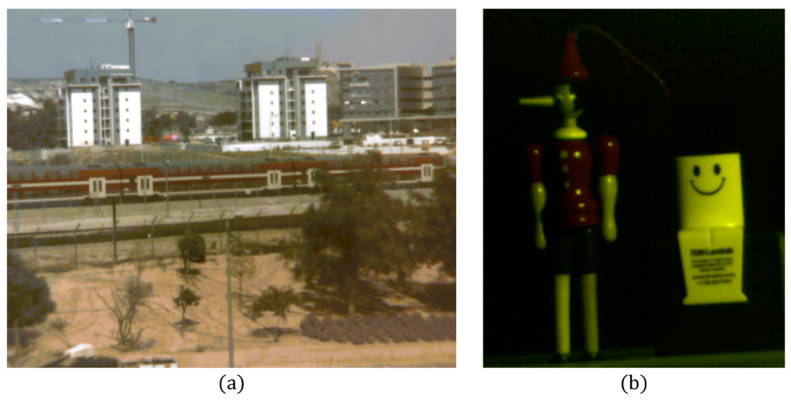
Staring mode reconstruction results with the dictionary of (**a**) outdoor and (**b**) indoor HS images taken with CS-MUSI camera. The figures show RGB representation of the reconstructed HS datacube.

The basic imaging mode of the CS-MUSI camera is the staring mode, so that the camera and scene do not move. Scanning mode acquisition is described in the next subsection. In staring mode, each spectrally multiplexed shot covers the same field-of-view (FOV) and, by taking *M* shots, a compressed HS datacube is captured. Figure 7, Figure 8 and Figure 9 demonstrate the reconstruction of spectral images (HS and ultra-spectral images) attained by the CS-MUSI camera in staring mode. Figure 7 presents the results of an experiment where the emission spectra of three arrays of red, green and blue light sources (Thorlabs LIU001, LIU002 and LIU003 LED arrays) were imaged using the CS-MUSI camera. Figure 7a shows the image of the light sources captured by a standard RGB color camera. The imaging experiment was performed by capturing 32 spectrally multiplexed images containing 1024 × 1280 pixels. Figure 7b–e shows four captured images that represent four single grayscale frames from the spectrally-compressed measurements. The images show the total optical intensity that has passed through the LC phase retarder and was collected by the sensor array at a given shot with a given LC voltage. From the captured data, a window of 700 × 700 pixels was used in the reconstruction process. Using the TwIST solver [33] and orthogonal Daubechies-5 wavelet as the sparsifying operator, a HS datacube with 391 spectral bands (410–800 nm) was reconstructed, yielding a compression ratio of about 12:1. Figure 7f presents a pseudo-color image obtained by projecting the reconstructed HS datacube onto the RGB space. Figure 7g–i displays three images from the reconstructed datacube at different wavelengths (460 nm, 520 nm and 650 nm). Figure 7k–m demonstrate spectrum reconstruction for three points in the HS datacube and a comparison to the measured spectra of the three respective LEDs with a commercial grating-based spectrometer. The reconstruction PSNR is 32.4 dB, 34.8 dB and 27.9 dB for the blue, green and red LED points, respectively.

Figure 8 presents an indoor scene where six different markers are imaged using the CS-MUSI camera. The imaging experiment was performed by capturing 100 spectrally multiplexed images containing 1024 × 1280 pixels. From the captured data, a window of 800 × 900 pixels was used in the reconstruction process. Using the TwIST solver [33] and orthogonal Daubechies-5 wavelet as the sparsifying operator, a HS datacube with 1171 spectral bands (410–800 nm) was restored, yielding a compression ratio of almost 12:1. Figure 8a presents a pseudo-color image obtained by projecting the reconstructed HS datacube onto the RGB space. Figure 8b displays four images from the reconstructed datacube at different wavelengths (470 nm, 530 nm, 580 nm and 630 nm).

The quality and time expenditure of reconstructed HS images depends significantly on the sparsifying operator. We found that by using a learned dictionary as the sparsifying operator [40] the quality and time improves. Figure 9 illustrates the reconstruction of HS images attained by the CS-MUSI camera using a spectral dictionary as the sparsifying operator (Section 3.1). The size of this dictionary was $N_{d}=1000$ atoms and was computed by using $N_{S}>$ 100,000 spectrum exemplars taken form a large database of HS images [42] and from a library of different spectra [40,43]. The HS images reconstructed from the CS-MUSI camera are in the range of 500 nm to 700 nm with 579 spectral bands, and were reconstructed from only 32 measurements, thus yielding a compression ratio of approximately 18:1. Figure 9a,b shows an RGB representation of the reconstructed HS images of outdoor and indoor scenes, respectively.

### 4.3. Scanning Mode

**Figure 10:**
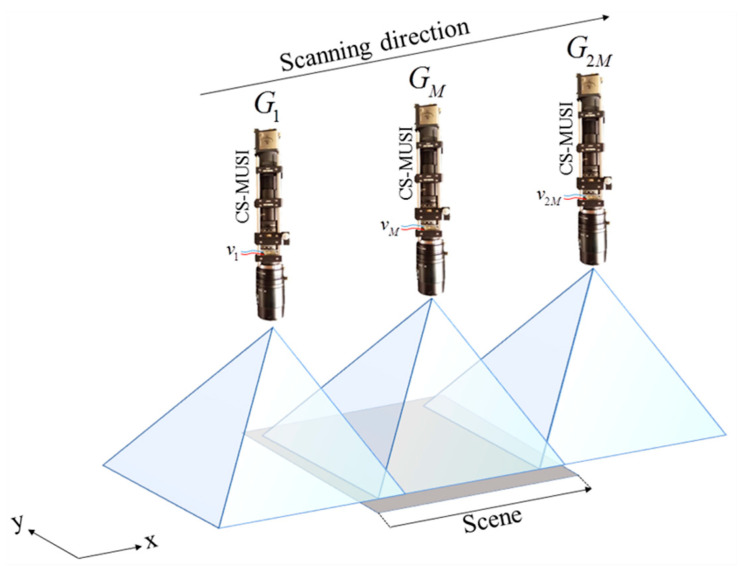
CS-MUSI camera along-track scanning. Each shot of the CS-MUSI camera, $G_{i}$, captures a shifted scene with a different LC spectral transmission (which depends on the voltage $v_{i}$).

The CS-MUSI camera can also be applied in a mode where the camera, the scene, or objects in the scene, are not stationary [44]. Such scenarios include microscope applications with moving cells or scanning platforms, and airborne and remote sensing systems. By capturing a sequence of spectrally multiplexed shots and tracking the object, it is possible to reconstruct HS data by an appropriate registration. It is required that the object appears in *M* shots. For example, in the case of along-track scanning [44] (Figure 10) the CS-MUSI camera needs 2*M* measurements in order to capture a scene of the size of the camera FOV. A second requirement is image registration, since the FOV of each shot is slightly different. As a result, before solving Equation (4) it is necessary to register all the measured images along a common spatial grid. This can be done with one of the many available algorithms [45,46,47].

**Figure 11:**
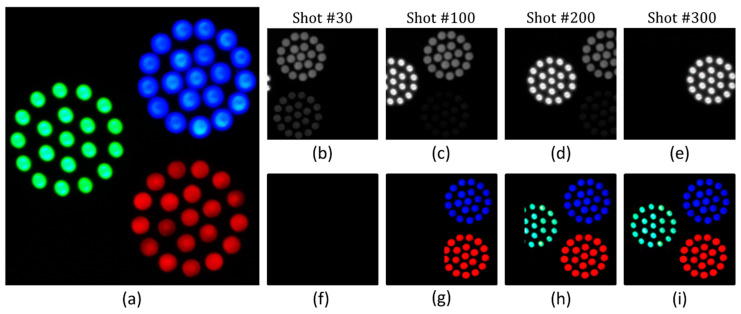
Scanning mode (Figure 10) reconstruction result. (**a**) RGB color image of three LED arrays. (**b**–**e**) representative single exposure images (frame #30, #90, #150 and #300, respectively) and (**f**–**i**) the RGB representation of the reconstructed HS image up to the appropriate column.

Figure 11 shows experimental results that demonstrate the ability to reconstruct HS images in a scanning mode. In this experiment, the scanning is along-track [44], which is similar to the push broom scanning technique [3]. The spectral multiplexed imaging acquisition process was conducted while the CS-MUSI camera was moving in front of three arrays of LED light sources (Thorlabs LIU001, LIU002, and LIU003) (Figure 11a). While the CS-MUSI camera was moving, a set of 100 voltages between 0 V and 10 V was applied repetitively. Figure 11b–e presents four representative spectrally multiplexed intensity measurements (shot #30, #100, #150 and #300, respectively) from a total of 300 measurements (Supplementary Materials). These images illustrated the along-track scanning of the camera from right to left, where in the first shot only part of the blue and red LEDs appears in the spectral multiplexed image, and in the last shot only the green LED appears.

Since the reconstruction was performed column by column [48], the reconstruction process can start before the scanning process is completed. Once a column is measured in *M* = 100 shots it can be reconstructed. Figure 11f–i shows an RGB representation of the reconstructed HS images up to different shot numbers. It can be noticed that at 30 shots (Figure 11f), no image column can be reconstructed as the total number of shots is smaller than *M* and no object column has enough measurements in order to be reconstructed. However, after *M* = 100 shots, some of the column images can be reconstructed after they have been measured *M* times. In this example, the HS image was reconstructed using the SpaRSA solver [35] and orthogonal Daubechies-4 wavelet as the sparsifying operator. From the 100 shots of each column, a HS image with 1171 spectral bands (410–800 nm) was restored, yielding a compression ratio of almost 12:1.

## 5. 4D Imaging

Integrating the CS-MUSI camera with an appropriate 3D imaging technique enables achieving a four-dimensional (4D) camera that can efficiently capture 3D spatial images together with their spectral information [49,50,51,52]. Joint spectral and volumetric data can be very useful for object shape detection [51,52] and material classification in various engineering and medical applications. The CS approach facilitated the acquisition effort associated with the huge dimensionality of the 4D spectral-volumetric data.

**Figure 12:**
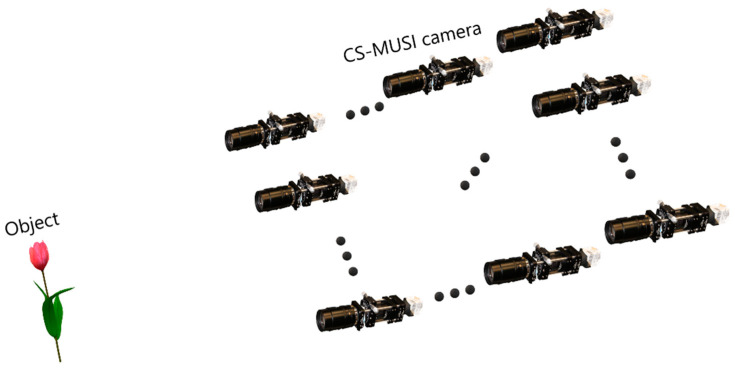
Compressive HS synthetic aperture InIm acquisition setup.

For 3D imaging we used Integral Imaging (InIm) [53,54,55,56], since its implementation is relatively simple. The first step of InIm is the acquisition of an actual 3D scene. In this step, multiple 2D images from slightly different perspectives are captured. Each of these images is called an elemental image (EI). Generally, the acquisition process can be implemented by a lenslet array (or pinhole array) or by synthetic aperture InIm. Synthetic aperture InIm (Figure 12) can be realized by an array of cameras distributed on the image plane, or by a single moving camera which moves perpendicularly to the system’s optical axis. Replacing the moving camera with our CS-MUSI camera enables capturing 3D spatial images together with their spectral information. By using the captured InIm data it is possible to synthesize depth maps, virtual perspectives and refocused images.

After the acquisition of the data and reconstruction of the spectral information from its compressed version, acquired with the moving CS-MUSI camera, the 3D image for each spectral channel (in terms of focal-stack) can be reconstructed numerically in different ways [49,56,57,58]. One of the most popular methods is based on back-projection, also known as shift-and-add. In the case of synthetic aperture InIm, the refocusing process can be performed as follows [49]:

(11) $$\tilde{I}\left(x,y,z,\lambda \right)=\frac{1}{o(x,y,z,\lambda )}\sum_{j=1}^{P}I_{j,\lambda }\left(x+Sh_{x,z,j},y+Sh_{y,z,j}\right),$$

where $\tilde{I}\left(x,y,z,\lambda \right)$ is the reconstructed data tesseract, $I_{j,\lambda }$ is the EI at wavelength λ showing the perspective from camera *j*. o(x,y,z,λ) is a normalizing matrix that normalizes each pixel in the image $\tilde{I}\left(x,y,z,\lambda \right)$ according to the number of EIs that the pixel appears in, and *P* is the overall number of EIs. $Sh_{x,z,j}$ and $Sh_{y,z,j}$ are the scaled size of the shifts in the horizontal and vertical directions of the CS-MUSI camera [49].

**Figure 13:**
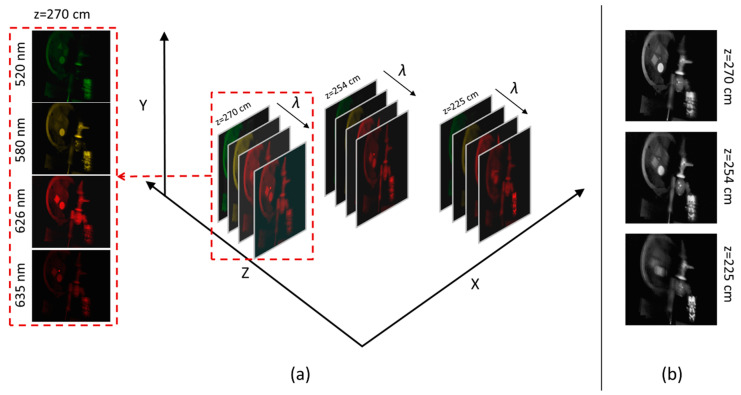
(**a**) 4D Spectro-Volumetric imaging. (**b**) Grayscale representation of HS images at three different depths (225 cm, 254 cm and 270 cm).

In the 4D imaging application, we acquired spectrally compressed images with our CS-MUSI camera from six perspectives, where in each perspective we captured 29 compressed images. Then, by using the TwIST solver [27], a HS datacube with 261 spectral bands (430–690 nm) was reconstructed. Next, we generated refocused images at different depths by using a shift-and-add algorithm [49]. The data can be ordered as a tesseract, as shown in Figure 13. Figure 13a demonstrates the reconstruction results at three selected depths for four selected wavelengths. The zoom on the HS datacube from the depth of 270 cm illustrates the spectral reconstruction quality, which can be observed from the fact that the laser beam appears clearly only at 635 nm, whereas it is completely filtered out in the other spectral bands (520 nm, 580 nm and 626 nm). From the grayscale images from Figure 13b it can be observed that the closest object was a green alien toy, whose best focus is at 225 cm; the Pinocchio toy’s best focus is at 254 cm and the best focus of the different colored shape objects and red laser is at 270 cm.

## 6. Target Detection

One key usage of spectral imagery is subpixel target detection, when an a priori known spectral signature is sought in each pixel of the spectral datacube. Previous researches dealt with target detection in the reconstructed domain [59], but in the case of the CS-MUSI camera, target detection can be applied in the compressed domain [60,61] since the CS-MUSI camera performs compression only in the spectral domain, without any spatial multiplexing. This yields a significant reduction of processing time and memory storage compared to non-compressing systems, of around an order of magnitude.

In order to test the subpixel target detection performance, we used the match filter (MF) algorithm [62], which can be derived by maximizing the SNR, or even by simply considering two hypotheses, as shown in Equation (12).

(12) $$\begin{array}{l} H_{0}:x=W\\ H_{1}:x=S+W \end{array}$$

where $H_{0}$ assumes that no target, $S={\left[s_{1},s_{2},\ldots ,s_{n}\right]}^{T}$, is present in the pixel and the pixel contains only background, $W∼N(0,\sigma^{2}I_{n\times n})$. $H_{1}$ assumes that both background and target are present in the pixel. For simplicity, both hypotheses are modeled as multidimensional Gaussian distributions.

By applying the log likelihood ratio test for $H_{0}$ and $H_{1}$ we may derive the MF, which is equal to:

(13) $$MF\left(x\right)=t^{T}\Gamma^{-1}\left(x-m\right),$$

where **x** is the pixel signature, **t** is the target spectral signature and **m** is the estimated background. Γ is the covariance matrix, which holds the statistics of the background and can be approximated using:

(14) $$\Gamma =\frac{1}{L}\sum \left(x-m\right){\left(x-m\right)}^{T},$$

where *L* is the number of pixels in the datacube. For a pixel that does not include the target, the MF takes the form of Equation (13). However, when the target is present we use an additive, as shown in Equation (15), and the MF takes the form of Equation (16):

(15) x′=x+pt,

(16) $$MF\left(x\prime \right)=t^{T}\Gamma^{-1}\left(x\prime -m\right),$$

where **x**’ is a pixel that contains the target and *p* is the ratio of the target present in the pixel.

In order to assess the algorithm’s performance, we adopt the performance metric that is mentioned in References [60,63]. Finally, we compare the Receiver Operating Characteristics (ROC) curve of the algorithm applied to an original HS datacube and the ROC curve of the same algorithm applied to a simulated compressed CS-MUSI datacube. The curve presents the positive detection vector as a function of false alarm probability, using the calculated value per threshold. The simulation is performed by applying a measured CS-MUSI sensing matrix, Φ, to each voxel of the HS datacube.

**Figure 14:**
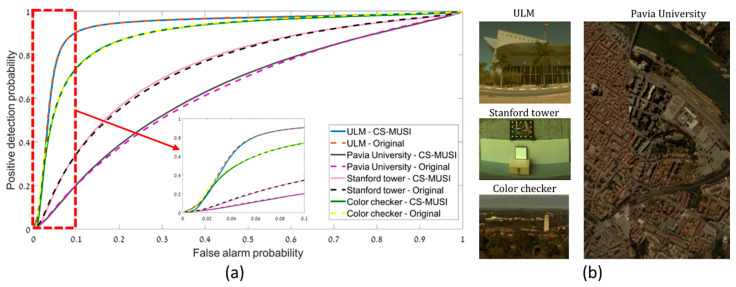
(**a**) Comparison of ROC curves for target detection in conventional (dotted lines) and CS-MUSI (solid lines) HS datacubes. (**b**) RGB representation of the four HS datacubes in the comparison [42,64,65].

Figure 14 presents results of CS-MUSI camera target detection performance. Figure 14a shows the comparison of ROC curves obtained from conventional HS datacubes (solid lines) to those captured with the CS-MUSI camera (dotted lines). Four pairs of ROC curves are presented for the four images shown in Figure 14b (from [42,64,65]). The compression ratio sets for the compressed datacube varied between 3.5:1 (lowest) and 25:1 (highest). From the ROC curves it can be seen that the performance is not degraded by the compression. Moreover, the detection speed in the compressed HS datacubes is increased due to lower computational complexity.

## 7. Discussion

We have overviewed an evaluation of the CS-MUSI camera [28] together with its different applications. We demonstrated reconstruction of HS images in the case where the camera and scene are stationary and for the case where the camera moves in the along-track direction. Furthermore, we demonstrated the ability to use the CS-MUSI camera for 4D spectral-volumetric imaging. Experiments in these scenarios and applications have demonstrated compressibility of at least an order of magnitude. Moreover, the results provide a spectral uncertainty of less than one nanometer, e.g., in Reference [28] we demonstrated an example that has a spectral localization accuracy of 0.44 ± 0.04 nm.

Additionally, we presented a remarkable property of the CS-MUSI camera showing that the target detection algorithm performs similarly with the CS-MUSI camera as with traditional HS systems, despite the fact that the CS-MUSI data is up to an order of magnitude less than that in conventional HS datacubes. Another important advantage of the CS-MUSI camera is its high optical throughput, due to the Fellgett’s multiplex advantage [66]. Furthermore, the CS-MUSI camera (Figure 5a) can be built with a small geometrical form and low weight by fabricating the LC cell to be attached to the sensor array.

It should be mentioned that the reviewed CS-MUSI camera has some limitations. The response time for a thick LC cell is relatively slow (in the order of a few seconds), which limits the acquisition frame rate. This limitation can be reduced by operating the cell in its transition state or with specially designed electronic functions, or by using faster LC structures such as ferroelectric LCs. Another limitation of the camera is the requirement for large computational resources for processing the data. This limitation can be mitigated by using parallel processing using GPU or multi-core CPU systems. Additionally, as with any HS processing algorithm, our reconstruction algorithm demands high memory capacity, since the storage of HS images can require gigabit sized memory. The additional memory requirements associated with the CS implementation are negligible compared to those of the HS data storage. From a theoretical CS point of view, the fact that there is no encoding in the spatial domain can be viewed as a limitation, since the compression obtained with the spectral encoding is lower than could have been theoretically obtained with encoding in all the three spatial-spectral domains [18]. On the other hand, the lack of spatial encoding makes it possible to maintain the full spatial resolution, allows parallel processing and facilitates the spectral imaging of moving objects.

The method of spectral multiplexing used in the CS-MUSI camera was carried out with a LC phase retarder as the spectral modulator. This method can be also realized with other spectral modulators [67]. In References [68,69], we used a modified Fabry-Perot resonator (mFPR) for spectrometry [68] and for imaging [69], which has a much faster response time compared to the LC cell. The method of spectral multiplexing can also be performed in parallel in order to achieve a snapshot HS camera. Lastly, in Reference [70] we presented a snapshot compressive HS camera that uses an array of mFPRs together with a lens array in order to acquire an array of spectrally multiplexed modulated sub-images.

## 8. Patents

In reference to the work presented here, a patent with the patent number US10036667 has been granted.

## Supplementary Materials

The following are available online at http://www.mdpi.com/2313-433X/5/1/3/s1, Video S1: Along-track scanning measurement. Each frame represents a multiplexed intensity measurement (total of 300 shots).
